# The impact of a rural sanitation programme on safe disposal of child faeces: a cluster randomised trial in Odisha, India

**DOI:** 10.1093/trstmh/trw043

**Published:** 2016-08-05

**Authors:** Matthew C. Freeman, Fiona Majorin, Sophie Boisson, Parimita Routray, Belen Torondel, Thomas Clasen

**Affiliations:** a Department of Environmental Health , Rollins School of Public Health, Emory University , 1518 Clifton Road NE , Atlanta , Georgia 30322 , USA; b Department of Disease Control, Faculty of Infectious and Tropical Disease Control , London School of Hygiene and Tropical Medicine , Keppel Street , London, WC1E 7HT , UK

**Keywords:** Child faeces, Diarrhoea, Faecal exposure, India, Sanitation, WASH

## Abstract

**Background:**

Unsafe disposal of child faeces is persistent and may lead to considerable impact on the health of young children. Research is limited on the impact of sanitation or hygiene interventions to improve child faeces disposal practices.

**Methods:**

In the context of a randomised controlled trial to assess the health impact of a programme in Odisha, India, to promote rural sanitation under the Government of India's Total Sanitation Campaign, we explored whether the intervention affected the safe disposal of faeces of children under-5 years of age.

**Results:**

At baseline, 1.1% of households practised ‘safe’ disposal of child faeces, either disposing it in a toilet or by burial. The intervention increased safe disposal of child faeces to 10.4% in intervention households, compared to 3.1% in the control households (RR 3.34; 95% CI 1.99–5.59). This increase in safe disposal is attributable to increases in latrine presence in the intervention communities; the intervention did not change safe disposal practices above and beyond the increase in latrine coverage.

**Conclusions:**

The very modest increase in safe disposal, while statistically significant, is not likely to have consequential health benefit. To achieve open defecation free communities, sanitation interventions will need to develop behaviour change approaches to explicitly target safe disposal behaviours.

## Introduction

 Nearly 1 billion people still practise open defecation globally, and a further 1.4 billion use unimproved toilet facilities. ^1^ The problem is especially severe in India, where 44% of the population still practise open defecation and only 40% of the population use improved sanitation. ^1^ In response, the Government of India launched a series of initiatives, including the Total Sanitation Campaign (TSC) (1999–2012), Nirmal Bharat Abhiyan (2012–2014) and most recently Swachh Bharat Abhiyan. ^2 , 3^ While these programmes have been successful in expanding sanitation coverage, the use of these facilities has been found to be poor. ^4–7^ Despite evidence of the positive health impact of improved sanitation generally, ^8 , 9^ rigorous evaluation programmes implementing the TSC have shown no effect on diarrhoea, soil-transmitted helminth infection or nutritional status. ^4 , 7^

 Compounding the low use among adolescents and adults is that even among households with access to improved sanitation, the faeces of children may not end up in the latrine. ^10^ In the latest demographic health survey in India, just 20% of child faeces ended in latrines—either the child defecated in the latrine, or it was placed there by a caregiver—the last time the child defecated. ^11^ Less than 1% was buried, a method currently characterised by the WHO/UNICEF Joint Monitoring Programme on Water Supply and Sanitation (JMP) as safe disposal. ^12^ However, a recent expert review deemed burial to be unsafe because of the thought among others that burial sites could be near the home and children's play areas and that the practice would not be acceptable for adults. ^13^ In children under 3 years, 16% of faeces were disposed of in any sanitation facility, ^14^ about half of which (9%) were improved facilities. ^15^ In a cross-sectional study in rural Odisha, India, among households with a latrine in villages households with a latrine in villages where the TSC had been implemented at least 3 years before, less than a quarter of the children's faeces ended up in a latrine. ^10^

 Despite the common perception that they are less ‘unclean’ or harmful than those of adults, ^16^ child faeces may present more of a health risk. Young children have the highest incidence of enteric infections ^17^ and are more likely to have pathogens in their stools. ^18^ A review in 2004 found that risky practices related to child faeces disposal resulted in a 23% increase in the risk of diarrhoea. ^16^ A 2016 study in Indonesia using data from demographic and health surveys, found unsafe disposal of child faeces to be strongly associated with child diarrhoea. ^19^ Children tend to defecate in areas where other children ^20^ and animals, such as dogs, ^21^ may come in contact with the faeces. Since children spend considerable time exploring their environment and practise behaviours such as geophagia, ^22^ they are more exposed to the contaminated soil, a potential risk factor for environmental enteropathy and stunting. ^23^

In this paper we report on whether the implementation of the TSC among rural households in Puri District in the State of Odisha impacted child faeces disposal behaviours. We also explored whether demographic or other characteristics of the study population predict safe disposal practices.

## Methods

### Background

 This study was nested within a village cluster-randomised controlled trial in Odisha between 2010 and 2013. Details of the study have been published elsewhere, including the study design, ^24^ process evaluation ^25^ and main health impacts. ^4^ Briefly, 100 villages selected to receive the TSC were randomised following baseline: half to receive a standard intervention delivered by WaterAid—an international non-governmental organisation—and its implementing partners, and the other half to serve as controls. A government subsidy covered most of the costs of materials and masonary support to construct a pour flush pit latrine with 1.5 m walls and a door. Householders were required to contribute labour, such as digging the pit, and if they wished to raise the latrine walls then they provided sand and bricks or stone for the walls. Although the intervention included mobilisers to encourage householders to participate, it did not include any significant behaviour change messages about toilet use, and no specific child faeces disposal behaviour change component. The baseline, conducted in the last quarter of 2010, showed latrine coverage in subsequently randomised to intervention and control groups of 9% and 8%, respectively. By March 2012, one year after the start of construction activities, 63% of households in the intervention communities had complete latrines compared to 12% in control villages. ^25^

### Data collection

 Data on child faeces disposal practices were collected at baseline (October 2010) and endline (October 2013) as part of a survey administered to households in both the intervention and control villages by a separate team that conducted health surveillance. ^26^ We included all villages chosen for the randomised trial and surveyed all households enrolled under health surveillance. A structured questionnaire was conducted in Oriya by enumerators trained in research ethics. Maternal heads of household were asked about the walking ability of their youngest child under 5 years old. Caregivers were asked about where that child typically defecated, and if the child did not defecate in the toilet, what they did, if anything, to dispose of the faeces. Additional demographic information was recorded, as well as water, sanitation and hygiene conditions at the home. 

### Data management and analysis

 Data were recorded on paper surveys and entered using EpiData 3.1 (EpiData Association, Odense, Denmark). Data were cleaned and analysed using STATA v. 13 (StataCorp, College Station, TX, USA). We compared child defecation behaviours using generalised estimating equations to calculate risk ratios, and accounted for clustering at the village level using an exchangeable correlation matrix. We analysed ambulatory and non-ambulatory children separately, since the child's ability to walk is a likely indicator of toilet use. ^5 , 27^ We used data collected during baseline to assess associations with safe disposal practices at endline. Disposal was considered ‘safe’ only if the faeces ended up in the latrine either because a child defecated in a latrine, or it was placed there by a caregiver or it was buried. ^28^ Models covariates were determined a priori and included the gender of the household head, if the main source of drinking water for the household was ‘improved’ as defined by the JMP, ^29^ if the water source was located in the compound, an observed household toilet in the compound, and an observed place for handwashing that included water and soap. Education of the household head was coded and categorised into ‘no or some primary education’ and ‘completed at least primary education.’ For assessments at endline, we included the intervention as a covariate. A wealth score was derived using principal component analysis from an asset index that included standardised variables. ^30 , 31^ However, the score accounted for only 13% of the total variation and failed to converge in models, so it was dropped from multivariable models. 

## Results

### Study participants

During the baseline survey, data from two villages were lost due to data collection error. Within 98 villages with available data, 7872 households were surveyed, of which 1958 were eligible for the study and consented: 1831 households had a child under 4 years of age and 288 had women in the third trimester of pregnancy (87 had both). An additional six households were eligible for the study but did not consent to the survey. We analysed a total of 1816 households with complete data. During the follow-up survey to assess child faeces disposal, we interviewed 2563 female caregivers. Among 2463 households interviewed with complete data, only 1780 (72%) with a child under 5 years old and complete data were analysed. In our assessment of determinants of faecal exposure behaviours at endline, we included only households that could be matched for both data collection rounds with children under 5 years old at endline (n=1092).

### Baseline disposal practices

At baseline, 45/1816 (2.5%) had a movable potty for use by children and 180/1816 (9.9%) had toilets. Few mothers (78/1816; 4.3%) reported that their youngest child defecated in a nappy, in a toilet (11/1816; 0.6%) or a potty (3/1816; 0.2%); the remainder reported defecation on the ground (1472/1816; 81.1%) or in their clothes (222/1816; 12.2%), and 30/1816 (1.7%) did not know. Among those whose child did not defecate in the toilet, 6/1805 (0.3%) placed it in the latrine, while 650/1805 (36.0%) put the faeces in the garbage or compost pit and 3/1805 (0.2%) buried it. The remaining left it out or did not move the faeces. As a result, 20/1816 (1.1%) practised safe disposal of child faeces; an equivalent number in the intervention (8/886; 0.9%) and control (9/930; 1.0%), p=0.89. At the time of this data collection, burial was considered ‘safe’ disposal. Using this definition of ‘safe’, 51/886 (5.8%) households in the intervention practised safe disposal, compared to 46/930 (5.0%) households in the control (p=0.43).

### Endline disposal practices

 At endline, among households with children under 5 years, 634/970 (65.4%) in the intervention group had an observed toilet compared to 153/810 (18.9%) in the control group. Among households with children under 5 years, 92/970 (9.5%) of intervention households had children that defecated in the toilet compared to control 22/810 (2.8%) households (RR 3.45; 95% CI 1.99–6.00; Table 1 ). The percent of caregivers that reported that the youngest child less than 5 years old defecated within the compound, was comparable between intervention (740/960; 77.1%) and control (623/800; 77.9%) households, but those that left the compound for open defecation, perhaps accompanied by the mother or an older sibling, was (155/800; 19.4%) in controls and (128/960; 13.3%) in the intervention households. Few households where children did not defecate in the toilet reported disposing of the faeces in the toilet in either the intervention (9/960; 0.9%) or control households (3/800; 0.3%); only one household buried the faeces. In the intervention villages, households with children under 5 were 3.3 times more likely to practise safe disposal of child faeces of their youngest child compared to households in control communities (RR 3.34; 95% CI 1.99–5.59). 

**Table 1.** Child defecation and faeces disposal practices at follow-up

	Intervention		Control		RR (95% CI) ^a^	p value
	n	*%*	n	*%*		
Any child	n=970		n=810			
Defecation						
In toilet ^b^	92	9.5	22	2.7	3.45 (1.99–6.00)	<0.001
In house or in compound	740	77.1	623	77.9	ref ^d^	NA ^e^
Outside compound	128	13.3	155	19.4	ref	NA
Disposal						
Safe disposal ^c^	102	10.5	25	3.1	3.34 (1.99–5.59)	<0.001
Thrown in latrine	9	0.9	3	0.3	- ^c^	NA
Buried	1	0.1	0	0.0	- ^c^	NA
Unsafe disposal						
Left where defecation occurred (left in open)	87	9.1	98	11.1	ref	NA
Thrown away inside compound	152	15.8	103	11.7	ref	NA
Thrown away outside compound	521	54.3	475	54.0	ref	NA
Washed away	63	6.6	56	6.4	ref	NA
Ambulatory children	n=770		n=642			
Safe faeces disposal	97	12.6	23	3.6	3.50 (2.06–5.94)	<0.001
Non-ambulatory children	n=195		n=168			
Safe faeces disposal	3	1.5	2	1.2	1.30 (0.22–7.67)	NS ^f^
Among those with a toilet						
Safe faeces disposal	98	15.5	22	14.4	1.10 (0.65–1.82)	NS

^a^ Risk ratios (RR) and 95% CI calculated using generalised estimating equations (GEE), with standard errors adjusted for clustering at the village level.

^b^ Comparison between defecation in toilet and defecation elsewhere.

^c^ Comparison between safe disposal in toilet (either defecation or disposal in toilet) or buried and all other unsafe disposal behaviors.

^d^ Ref refers to the referent groups for the risk ratio calculations.

^e^ NA: not applicable as p-values are only calculated for a single estimate of the risk ratio.

^f^ NS: not significant at p<0.05.

Parents reported that 79.3% of children (1412/1780) were ambulatory and 363/1780 (20.4%) could not walk; data on mobility was missing for 5/1780 (0.3%). Among children under 5 who were not ambulatory, few households practised safe disposal of child faeces. Only 3/195 (1.5%) households in the intervention group and 2/168 (1.2%) in the control group practised safe disposal of child faeces (RR 1.30; 95% CI 0.22–7.67). Among children under 5 who were ambulatory in the intervention households, 97/770 (12.6%) practised safe disposal practices, which primarily consisted of use of the latrine (91; 11.9%). Safe disposal in intervention communities was 3.5 times higher compared to the control communities where 23/642 (3.6%) had safe faeces disposal (RR 3.50; 95% CI 2.06–5.94).

Of the 126 households that practised safe disposal of child faeces, 120 (95.2%) had a toilet. For the subset of the households with a toilet, the percentage of households that had safe child faeces disposal practices—typically use of the toilet by an ambulatory child—was similar between households within the intervention (98/634; 15.5%) and control (22/153; 14.4%) villages (RR 1.10; 95% CI 0.66–1.82). In other words, those in the intervention communities with a toilet were no more likely than those with toilets in the control to safely dispose of the child faeces.

### Associations with safe disposal practices at endline

 Intervention status alone was a strong predictor of safe faeces disposal practices (RR 3.26; 95% CI 1.85–5.76, data not shown). However, in multivariable analysis, intervention status was not associated with the safe disposal of child faeces (RR 1.11; 95% CI 0.67–1.82; Table 2 ). The presence of a toilet was strongly associated with safe disposal (RR 31.5; 95% CI 9.45–104), as was education of the household head (RR 1.82; 95% CI 1.07–3.11); water in the compound was weakly associated with safe faeces disposal (RR 1.45; 95% CI 0.95–2.22). An improved drinking water source was associated with poorer safe disposal practices (RR 0.57; 95% CI 0.35–0.95). 

**Table 2.** Adjusted associations between household demographics and conditions and safe disposal of child faeces

	Endline			
	Unsafe disposal (n=997)	Safe disposal (n=95)	RR (95% CI) ^a^	*p*
Male head of household	942 (94.5%)	92 (96.8%)	0.70 (0.24–2.07)	NS
Household head completed at least primary school	685 (68.7%)	81 (85.3%)	1.82 (1.07–3.11)	0.03
Drinking water source is improved	852 (85.5%)	79 (83.2%)	0.57 (0.35–0.95)	0.03
Water in compound	268 (26.9%)	48 (50.3%)	1.45 (0.95–2.22)	NS
Access to a toilet in compound	376 (37.7%)	92 (96.8%)	31.5 (9.45–104)	<0.001
Presence of place for handwashing with water and soap	105 (10.5%)	26 (27.4%)	1.25 (0.80–1.94)	NS
Intervention	511 (51.2%)	75 (79.0%)	1.11 (0.67–1.82)	NS

^a^ Risk ratios (RR) and 95% confidence intervals calculated using generalized estimating equations (GEE), with standard errors adjusted for clustering at the village level.

NS: not significant at p<0.05.

## Discussion

 Unsafe disposal of child faeces is persistent, ^32^ yet few sanitation and hygiene programmes, even those that focus on promotion of open defecation free communities, have focused on safe disposal of child faeces. However, a considerable proportion of both symptomatic and asymptomatic children shed pathogens in their stool, ^33^ and contamination of the environment with child faeces represents a considerable potential risk to health. In accordance with the JMP definition, we defined safe disposal as depositing faeces in a latrine or burying. However, this does not guarantee that the faeces will not contaminate the environment if waste stream from the latrines is not properly managed. In addition, a recent Delphi consultation concluded that burial should not constitute ‘safe’ disposal, due to the proximity of burial sites to children's play areas and that a similar practice would not be appropriate for adults. ^13^ Practices like disposal with a solid waste service may be a better option for households without toilets than just leaving it in the yard or washing it into a water source, though while this approach would not pose immediate risks to the household directly, it would not be considered safe due to the risk of environmental contamination. 

We explored whether an intervention in Odisha to promote rural sanitation under the Government of India's TSC impacted the safe disposal of faeces of children under 5 years, within the context of a randomised controlled trial to assess health impact. Safe disposal was rare, with only 1% of study households following the practice prior to the introduction of the intervention. This low level was not surprising, since open defecation was common in this population at baseline, with less than 10% of study households having a latrine that would have allowed them to safely dispose of their children's faeces.

 While the intervention increased latrine coverage in study villages to 63% (65% in the full trial population ^25^ ), this may be insufficient to yield health gains even assuming full use, including for disposal of child faeces. However, we found that among intervention households at endline, only 10.4% of mothers reported that child faeces ended up safely disposed in latrines. Moreover, this change from baseline in safe disposal was solely due to an increase in the number of households with latrines; at endline, intervention households with toilets were no more likely than those with toilets in the control to safely dispose of the child faeces. Thus, beyond increasing latrine coverage, we did not find evidence that the intervention had any impact on safe disposal of child faeces. 

 While the increase in child faeces disposal over baseline was substantial, evidence suggests that it would be insufficient to result in health gains. Community latrine coverage may need to exceed 75% in order to see significant reductions in diarrhoeal disease. ^34^ Improvements in community defecation practices may result in commensurate declines in childhood stunting, but the effects of the communities practices exceeds the influence of the household practices. ^35^

 Access to a latrine is a necessary but clearly insufficient condition of practising safe disposal of child faeces; most households with latrines still do not follow the practice. At endline, safe disposal was associated weakly with having a water point within the compound, but inversely related to having an ‘improved’ water source for drinking. Having a water point within the compound is strongly related to household water availability, which is strongly linked with latrine usage in Odisha ^27^ and may explain the relationship with safe disposal of child faeces. However, having an ‘improved’ drinking water source is not indicative of increased water availability, potentially explaining the counter intuitive results. Other studies have found safe disposal to be associated with household wealth, mother's education, child age, years of latrine ownership, caregiver age, consistency of adult latrine use and presence of child faeces management tools in the latrine. ^36 , 37^ Our results are consistent with other studies that report poor and inconsistent use of latrines for disposal of child faeces. ^10 , 36 , 37^ They are also consistent with other findings regarding sub-optimal use of latrines constructed under the TSC, ^4 , 5 , 7^ which did not include explicit behaviour change elements that focused on either toilet use or safe disposal of child faeces. 

 Challenges in achieving correct, consistent use of latrines in India have been documented elsewhere. ^27 , 38^ However, there are additional obstacles to overcome to increase safe disposal of child faeces. Perhaps chief among these is the perception that child faeces do not present a risk to human health. ^27^ This knowledge barrier is consistent with findings by others and us that safe disposal is associated with parents’ education and caregiver awareness. ^23^ On the other hand, research has suggested that in the area of personal hygiene, the development of healthfully habits, such as handwashing after defecation, is not motivated by knowledge but on other motivators or structural facilitators of behaviour change. ^39–41^ Recent research in Cambodia suggests a need for a more comprehensive understanding of the barriers to safe disposal of child faeces, and for specific hardware interventions, such as reusable diapers, child-friendly potties and latrine seats that offer child safety. ^37^

There is increasing acknowledgement that interventions that increase latrine coverage do not necessarily ensure latrine use—a clear condition to achieving health gains from sanitation. To optimise health impacts, it is also important that such use also includes the safe disposal of child faeces. Both require specific efforts that are informed by a deeper understanding of barriers to adopting the targeted behaviours and that are supported by policies that encourage programmatic efforts to overcome them.

 There were several limitations to this study. First, we relied on caregiver self-report for our key outcomes, a potential source of bias in the context of an intervention study where treatment status was not blinded. Direct observation may be more objective, but are also prone to bias. ^42^ Second, we only assessed behaviour at a single time point. Our pilot data collected among a subset of households in October 2013 found safe disposal of child faeces in the intervention group of 26%, and 5% in the control households (data not shown). Additional data collection at a different season or time following implementation may have revealed different findings. Third, the intervention did not include any specific behaviour change component, let alone a focus on disposal of child faeces. The purpose of this study was to assess the impact of the TSC as it was implemented. Future research should focus on optimising the effectiveness of sanitation and hygiene interventions to improve child faeces disposal practices. 

### Conclusions

 We found that while safe disposal of child faeces increased considerably from baseline, and was significantly different between intervention and control, the increase in safe disposal was directly related to commensurate increases in latrine presence in the intervention communities. The intervention did not substantially change behaviours above and beyond the expected change associated with greater latrine coverage. The very modest increase in safe disposal, while statistically significant, is not likely to have consequential health benefit as it likely did not mitigate community or household exposure to faecal pathogens. ^35^ To achieve open defecation free communities, sanitation interventions will need to develop behaviour change approaches to explicitly target safe child faeces behaviors. 
